# Construction and characterization of rectal cancer‐related lncRNA‐mRNA ceRNA network reveals prognostic biomarkers in rectal cancer

**DOI:** 10.1049/syb2.12035

**Published:** 2021-10-06

**Authors:** Guoying Cai, Meifei Sun, Xinrong Li, Junquan Zhu

**Affiliations:** ^1^ Department of Integrative Medicine & Medical Oncology Shengzhou People's Hospital (the First Affiliated Hospital of Zhejiang University, Shengzhou Branch) Shengzhou Zhejiang China

**Keywords:** lncRNA, network, prognosis biomarkers, rectal cancer, transcription factor

## Abstract

Rectal cancer is an important cause of cancer‐related deaths worldwide. In this study, the differentially expressed (DE) lncRNAs/mRNAs were first identified and the correlation level between DE lncRNAs and mRNAs were calculated. The results showed that genes of highly correlated lncRNA‐mRNA pairs presented strong prognosis effects, such as *GPM6A*, *METTL24*, *SCN7A*, *HAND2‐AS1* and *PDZRN4*. Then, the rectal cancer‐related lncRNA‐mRNA network was constructed based on the ceRNA theory. Topological analysis of the network revealed that the network was maintained by hub nodes and a hub subnetwork was constructed, including the hub lncRNA MIR143HG and MBNL1‐SA1. Further analysis indicated that the hub subnetwork was highly related to cancer pathways, such as ‘Focal adhesion’ and ‘Wnt signalling pathway’. Hub subnetwork also had significant prognosis capability. A closed lncRNA‐mRNA module was identified by bilateral network clustering. Genes in modules also showed high prognosis effects. Finally, a core lncRNA‐TF crosstalk network was identified to uncover the crosstalk and regulatory mechanisms of lncRNAs and TFs by integrating ceRNA crosstalks and TF binding affinities. Some core genes, such as MEIS1, GLI3 and HAND2‐AS1 were considered as the key regulators in tumourigenesis. Based on the authors’ comprehensive analysis, all these lncRNA‐mRNA crosstalks provided promising clues for biological prognosis of rectal cancer.

## 1 INTRODUCTION

Rectal cancer is a common malignant tumour in the gastrointestinal tract, the incidence of which is second only to gastric and oesophageal cancers [1]. Despite definite surgical treatment, rectal cancer is still associated with poor clinical outcomes and is prone to local recurrence and systemic metastasis [2]. With the improvement of surgical techniques and neoadjuvant treatment strategies, the local recurrence rate of rectal cancer has been decreased historically from 25% down to about 5%–10%. However, distant metastatic relapse has still not been reduced, which is the main cause of rectal cancer‐related death [3]. In the past few years, many studies have focussed on the research of rectal cancer. Crucial advancements have been made in the field of molecular biology and genomics, such as microsatellite instability (MSI)/stability (MSS) [4], RAS pathway‐related molecules [5] and human epidermal growth factor receptor‐2 (HER2) status [6], which had greatly contributed to optimize treatment and the diagnostic strategy of advanced rectal cancer, but new approaches are still needed to further reveal the mechanisms underlying the pathogenesis of rectal cancer. From the bioinformatics point of view, it is important to identify specific prognostic biomarkers for long‐term survival of rectal cancer patients.

Long non‐coding RNAs (lncRNAs) are the main focus of attention among non‐coding RNAs (ncRNAs), which modulate the biological behaviours of tumour cells [7]. LncRNAs play key roles in cancer‐related biological processes, such as proliferation, invasion and metastasis [8, 9]. Previous studies have revealed that lncRNAs were closely involved in rectal cancer. For example, three lncRNA signatures, including *lnc‐KLF7‐1*, *lnc‐MAB21L2‐1* and *LINC00324* were identified as ideal biomarkers for response prediction of neoadjuvant chemoradiotherapy in locally advanced rectal cancer [10]. LncRNA *LINC00461* was reported to mediate cisplatin resistance of rectal cancer [11]. LncRNA *PCAT1* rs2632159 SNP was suggested to be a potential biomarker for rectal cancer susceptibility [12]. In addition, lncRNAs have also been shown to function in multiple other aspects, ranging from chromatin remodelling to regulation of transcription and post‐transcription [13] by interaction with other molecules. Competitive endogenous RNA (ceRNA) is a popular hypothesis [14] to elucidate the complex relationship between lncRNA, miRNA and mRNA at the transcriptional level. That is, LncRNAs could competitively bind miRNAs to regulate the mRNA transcription, which indicates that a potential close association could be explored between the lncRNAs and mRNA with the shared miRNAs [15]. This kind of RNA interactions could significantly help understand lncRNA‐mRNA networks and have implications in the study of human cancers [16]. However, until now, few studies about the specific ceRNA regulatory mechanism between lncRNA, miRNA and mRNA in rectal cancer have been reported. The regulatory relationship between some key lncRNAs or mRNAs of the ceRNA network and even the crosstalk between lncRNAs and transcription factors (TFs) are less studied.

To solve the above problems, in this study, we identified differentially expressed (DE) lncRNAs and mRNAs in rectal cancer. The rectal cancer‐related lncRNA‐mRNA network was constructed by the DE lncRNAs/mRNAs paired on the basis of the ceRNA theory, which would elucidate novel molecular mechanisms involved in the initiation and progression of rectal cancer. Then, hub subnetwork was extracted from the initial network for focussing on some important lncRNAs/mRNAs that may be associated with rectal cancer. The local functional module was further identified to suggest that a group of lncRNAs and mRNAs with closer contact may be biomarkers to reveal potential molecular mechanisms of rectal cancer occurrence and development. In addition, a lncRNAs‐TFs network was established to display the crosstalk and regulatory mechanisms between lncRNAs and TFs. Finally, survival analysis was, respectively, used for important lncRNAs of the network, hub subnetwork and functional module for showing the effective prognosis value for rectal cancer.

## 2 MATERIALS AND METHODS

### 2.1 Data sets

We downloaded The Cancer Genome Atlas (TCGA) gene expression dataset of rectal cancer from UCSC XENA browser (https://xenabrowser.net/datapages/), which contained the transcript‐level expression profile of a total of 167 tumour samples and 10 adjacent non‐tumour samples. Based on the gene ID conversion that supported by GENCODE (https://www.gencodegenes.org/human/), transcripts with Ensembl IDs in the dataset were converted to lncRNAs and mRNAs with Gene Symbols. If multiple transcripts corresponded to one lncRNA/mRNA, the mean expression value of multiple transcripts was considered as expression value of the lncRNA/mRNA. Some genes with zero values in more than 30% samples were deleted. The remaining zero values were set to 0.1 based on a previous study [17]. Then, we standardized raw gene expression values by performing log2 transformation and obtained both lncRNA expression profile and mRNA expression profile of rectal cancer. For performing the following survival analysis, we also downloaded clinical information of the corresponding rectal cancer samples from UCSC XENA.

### 2.2 Methods

**FIGURE 1:**
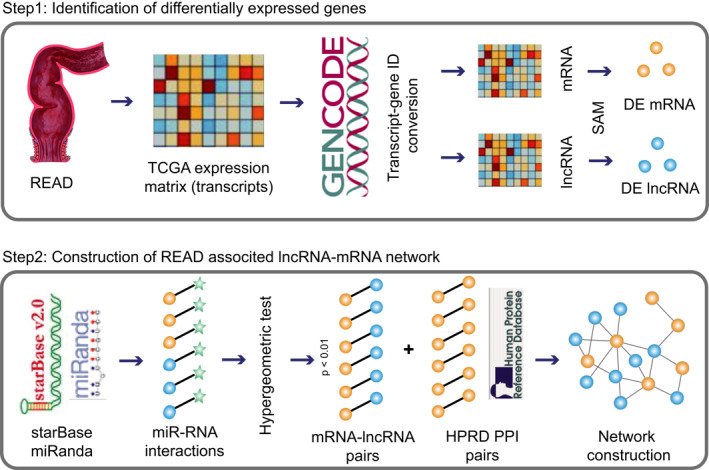
Pipeline of the construction of rectal cancer‐related lncRNA‐mRNA network. Step 1, the gene expression dataset of rectal cancer was downloaded from TCGA. Transcripts with Ensembl IDs in the dataset were converted to lncRNAs and mRNAs with Gene Symbols by GENCODE. SAM test was used to calculate rectal cancer‐related DE lncRNAs and mRNAs. Step 2, significant miRNA‐lncRNA interactions were identified by miRanda. All miRNA‐mRNA interactions were downloaded from starBase. Hypergeometric test and Pearson correlation test were used for identifying significant lncRNA‐mRNA ceRNA pairs. All the significant lncRNA‐mRNA ceRNA pairs and DE mRNA‐DE mRNA pairs from HPRD were integrated to construct a rectal cancer‐related lncRNA‐mRNA network

In this study, we constructed a rectal cancer‐related lncRNA‐mRNA network based on the differentially expressed (DE) lncRNAs/mRNAs and ceRNA theory (Figure 1). Then, we performed several following analyses, including network topology analysis, hub subnetwork extraction, module identification and survival analysis.

### 2.3 Calculate DE lncRNAs/mRNAs

Based on the lncRNA and mRNA expression profiles, we used Significance analysis of microarrays (SAM) test to calculate rectal cancer‐related DE lncRNAs and mRNAs under the threshold of 2‐fold change (FC) and *p*‐value <0.05.

### 2.4 Construct rectal cancer‐related lncRNA‐mRNA network

We constructed a rectal cancer‐associated lncRNA‐mRNA network based on the DE lncRNAs/mRNAs and ceRNA theory, as follows:

First, 423,975 miRNA‐mRNA interactions between 386 miRNAs and 13,861 mRNAs were downloaded from starBase [18]. Rectal cancer‐related DE mRNAs were mapped into these miRNA‐mRNA interactions for obtaining miRNA‐DE mRNA interactions. MiRNA‐DE lncRNA interactions were also obtained using the miRanda tools (default parameters) with the miRNA and DE lncRNA sequences as input data.

Second, the above miRNA‐DE mRNA interactions and miRNA‐DE lncRNA interactions were used for extracting the shared miRNAs between DE lncRNAs and DE mRNAs. Hypergeometric test was further applied for calculating the statistical significance of the number of the shared miRNAs. For each lncRNA and each mRNA, if the number of the shared miRNAs between them met the threshold of hypergeometric test *p*‐value <0.01, we could get a candidate lncRNA‐mRNA pair. The following formula was used to calculate hypergeometric test *p*‐value:

$$p-\text{value}=1-\sum_{i=0}^{r-1}\frac{(\begin{array}{l} t\\ i \end{array})\left(\begin{array}{l} m‐t\\ n‐i \end{array}\right)}{\left(\begin{array}{l} m\\ n \end{array}\right)}$$

where, *m* is the number of miRNAs in starBase, *n* is the number of miRNAs in the miRNA‐DE lncRNA interactions, *t* is the number of miRNAs in the miRNA‐DE mRNA interactions, and *r* is the number of the shared miRNAs between each lncRNA and each mRNA.

Based on ceRNA hypothesis, the ceRNA pairs should meet not only the statistical significance of hypergeometric test but also the expressional correlation. That is to say, the lncRNA and mRNA of each ceRNA pair should be correlated in terms of their expression. Therefore, next, we computed Pearson correlation coefficients (PCC) for the above candidate lncRNA‐mRNA pairs. Those candidate lncRNA‐mRNA pairs that met the threshold of PCC >0.6 were considered as significant lncRNA‐mRNA ceRNA pairs.

In order to maintain the integrity of the network, we mapped DE mRNAs into protein‐protein interactions that were downloaded from HPRD (http://www.hprd.org) and extracted DE mRNA‐DE mRNA pairs. We further integrated all the significant lncRNA‐mRNA ceRNA pairs and DE mRNA‐DE mRNA pairs for the construction of a rectal cancer‐related lncRNA‐mRNA network.

### 2.5 Analyse network topology features

We systematically analysed three most robust measures of network topology, including degree distribution, cluster coefficient and average path length. A network is a system of nodes and edges. There are many indicators to describe the importance of network nodes. The simplest one is the degree of nodes, which is equal to the number of direct neighbours of nodes [19]. Cluster coefficient represents the degree of aggregation of nodes in a network graph. In a particular network, nodes tend to form a set of tightly organized relationships if they have high cluster coefficient [20]. If the average clustering coefficient of a network is significantly higher than that of a random network generated by the same set of nodes, then the network could be considered a small world. Average path length is defined as the average number of steps along the shortest paths for all possible pairs of network nodes, which is a measure of the efficiency of information transport on a network [21].

### 2.6 Extract hub subnetwork from the lncRNA‐mRNA network

In a scale‐free network, the degrees of most nodes are small. However, the degrees of a small number of nodes are very large, which are called hub nodes. In a biological network, if known disease genes are hub nodes, then the disease information is more likely to spread around the network. Therefore, in this study, we chose top 10% lncRNAs and top 5% mRNAs with the larger degrees together as hub nodes. Then, a hub subnetwork was extracted from our rectal cancer‐related lncRNA‐mRNA network, which contained hub lncRNAs, hub mRNAs and the interactions between them.

### 2.7 Identify functional modules from the lncRNA‐mRNA network

Identifying important information from biomolecular network, such as functional modules, is a key problem in bioinformatics. The independence of various functional modules in biological systems is an important factor in maintaining biological stability [22]. In this study, we used the Molecular Complex Detection (MCODE) plug‐in in Cytoscape software to identify rectal cancer‐related functional modules from the lncRNA‐mRNA network. The MCODE algorithm clusters a given network by topology and finds densely connected regions based on graph‐theoretical analysis [23]. The criteria that we used for identifying rectal cancer‐related functional modules were as follows: MCODE scores >5, degree cut‐off = 2, node score cut‐off = 0.2, max depth = 100, and *k*‐score = 2.

### 2.8 Survival analysis

For identifying novel therapy targets for long‐term survival of rectal cancer patients, survival analysis was performed by constructing a risk score model. The risk score for each patient was computed by linear combination of the lncRNA/mRNA expression values weighted by the regression coefficient of univariate Cox regression analysis via ‘coxph’ in ‘survival’ package. Hazard ratio is also calculated by ‘coxph’. Here, we used all the TCGA READ dataset as the training set to yield the training parameters (regression coefficients). The following formula was used to calculate risk score:

$$\text{Risk}\;\text{Score}=\sum_{i=1}^{n}\beta_{i}\text{Exp}\left(i\right)$$

where *n* is the number of lncRNAs/mRNAs in gene set; *β*_*i*_ is the Cox regression coefficient of the lncRNA/mRNA from an independent gene set; and Exp(*i*) is the expression value of the lncRNA/mRNA in a corresponding patient.

The mean risk score was used as a cut‐off to classify patients into high‐risk group and low‐risk group. A Kaplan–Meier survival curve was performed for different groups of rectal cancer patients. The statistical significance was assessed by the log‐rank test with a threshold of *p* < 0.05.

## 3 RESULTS

### 3.1 Comprehensive analysis of lncRNA/mRNA expression dataset

**FIGURE 2:**
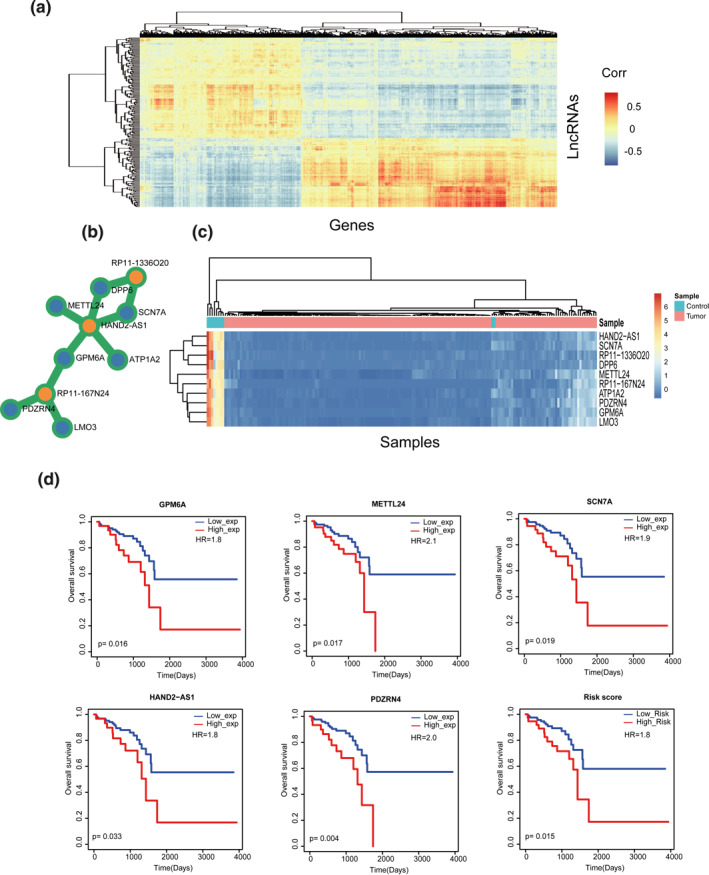
Comprehensive analysis of lncRNA/mRNA expression dataset. (a) DE lncRNAs and DE mRNAs were strongly expression correlated. The red colour indicates positive correlation and the blue colour indicates negative correlation. (b) 10 lncRNA‐mRNA pairs with the highest correlation scores formed a small network. The orange represents three DE lncRNAs and the blue represents seven DE mRNAs. (c) The heat map of hierarchical clustering for the 10 both DE and highly correlated nodes. (d) Kaplan–Meier survival curves of these core nodes

We obtained the standardized lncRNA expression profile and mRNA expression profile of rectal cancer, containing 167 tumour samples and 10 adjacent non‐tumour samples. SAM test with the threshold of 2‐FC and *p*‐value <0.05 was used to identify 206 DE lncRNAs and 2085 DE mRNAs. In order to perform the following in‐depth analysis, we computed the correlation between DE lncRNAs and DE mRNAs by PCC. The result of heat map showed that these DE lncRNAs and DE mRNAs were strongly expression correlated (Figure 2a). We chose 10 lncRNA‐mRNA pairs with the highest correlation scores and used the shared lncRNAs/mRNAs to form a small network. We found that this network consisted of three DE lncRNAs and seven DE mRNAs (Figure 2b). The three lncRNAs, including *HAND2‐AS1*, *RP11‐167N24* and *RP11‐1336O20* were all highly correlated with multiple DE mRNAs, which suggested the importance of the regulatory role of lncRNAs in the research of rectal cancer. We also chose top 20 lncRNA‐mRNA pairs with the highest correlation scores. The results also showed that the lncRNAs *HAND2‐AS1*, *RP11‐167N24* and *RP11‐1336O20* were the core regulators in READ (Supplementary Figure S1). We then clustered the expression of both these 10 DE and highly correlated nodes in our rectal cancer‐related 167 tumour samples and 10 adjacent non‐tumour samples. The result of hierarchical clustering showed that these 10 core nodes could clearly distinguish the samples (Figure 2c). Furthermore, we used these 10 core nodes for survival analysis to understand their impact on survival of rectal cancer patients. The results of Kaplan–Meier survival curves showed that high/low expression (more or less than median of expression value, respectively) of five nodes (*GPM6A*, *METTL24*, *SCN7A*, *HAND2‐AS1*, *PDZRN4*) could significantly clarify rectal cancer patients into two groups with different survival outcomes by the threshold of log‐rank test *p* < 0.05 (Figure 2d). Simultaneously, linear combination of expression values of these 10 core nodes weighted by the regression coefficient of univariate Cox regression was also calculated to distinguish patients into high‐risk group and low‐risk group by the median of risk scores. Then, the high‐risk group and low‐risk group of rectal cancer patients were significantly classified with different clinical outcomes (Figure 2d) by performing survival analysis. These results suggested that the DE lncRNAs have significant prognosis capabilities, which deserved further analysis.

### 3.2 Construction of the rectal cancer‐related lncRNA‐mRNA network

**FIGURE 3:**
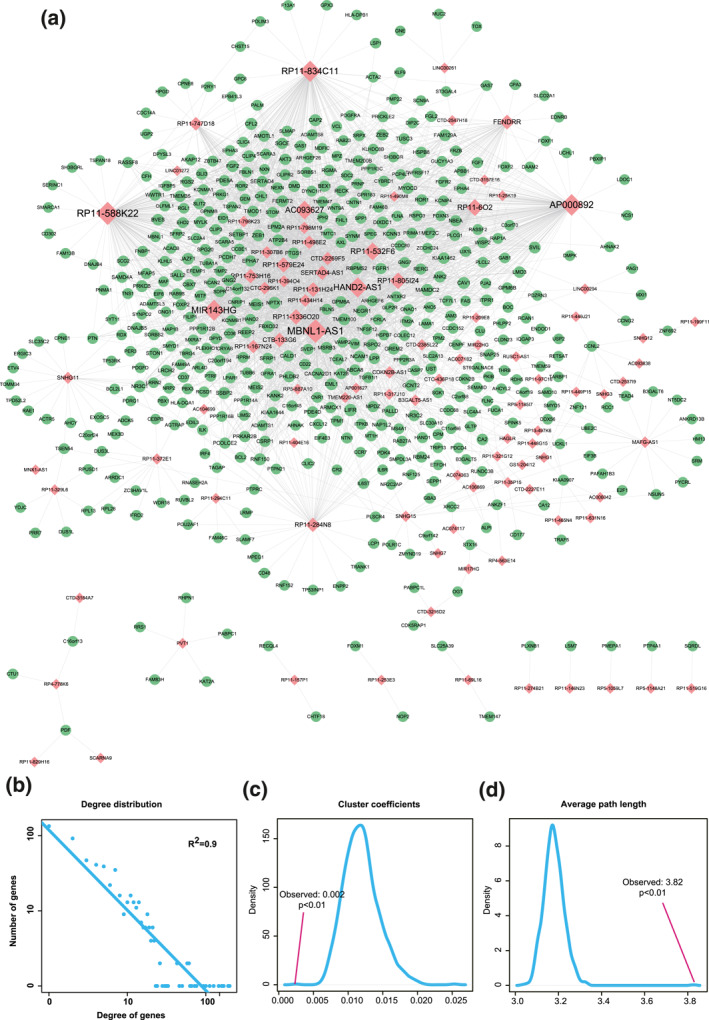
Topology features of the rectal cancer‐related lncRNA‐mRNA network. (a) Network visualization. Red nodes represent lncRNAs and green nodes represent mRNAs. Node size represents degree of node. (b) Degree distribution of the network. All nodes followed a power‐law distribution. (c) Average cluster coefficient of the real network was significantly larger than that of 1000 random networks. (d) Average path length of the real network was significantly shorter than that of 1000 random networks

Rectal cancer‐related DE mRNAs were mapped into miRNA‐mRNA interactions from starBase for obtaining 40,850 miRNA‐DE mRNA interactions. The miRanda tools identified significant 7265 miRNA‐DE lncRNA interactions between miRNAs and DE lncRNAs. Then, based on the ceRNA theory, 2660 significant lncRNA‐mRNA ceRNA pairs were identified with the hypergeometric test *p*‐value <0.01 and PCC >0.6. By further integrating DE mRNA‐DE mRNA pairs from HPRD, we constructed a rectal cancer‐related lncRNA‐mRNA network. This network consisted of 96 lncRNAs, 484 mRNAs and 2738 edges between them (Figure 3a). In order to understand the overall structural characteristics of the network, three most robust network topology features were systematically analysed. First, we counted the degrees of all the nodes in the network and found that most nodes had small degrees but a few nodes had very large degrees. We performed degree distribution to the whole network and found all nodes in the network following power law distribution (Figure 3b, *R* ^2^ = 0.9). Second, we computed cluster coefficients of the real lncRNA‐mRNA network and 1000 random networks generated by the same degrees of nodes. The results showed that the average cluster coefficient of the real network was significantly larger than that of the random networks (Figure 3c, *p* < 0.01). Actually, the network with larger average cluster coefficient usually had modular structures with smaller average distances between nodes. Thus, we further calculated the average path length of the lncRNA‐mRNA network and found that the average path length of real network was significantly shorter than that of random networks (Figure 3d, 1000 random networks with the same degrees of nodes, *p* < 0.01). It demonstrated that the rectal cancer‐related lncRNA‐mRNA network was a small world network where every node was connected to the other nodes through a very short path.

### 3.3 Extraction of a rectal cancer‐related hub subnetwork

**FIGURE 4:**
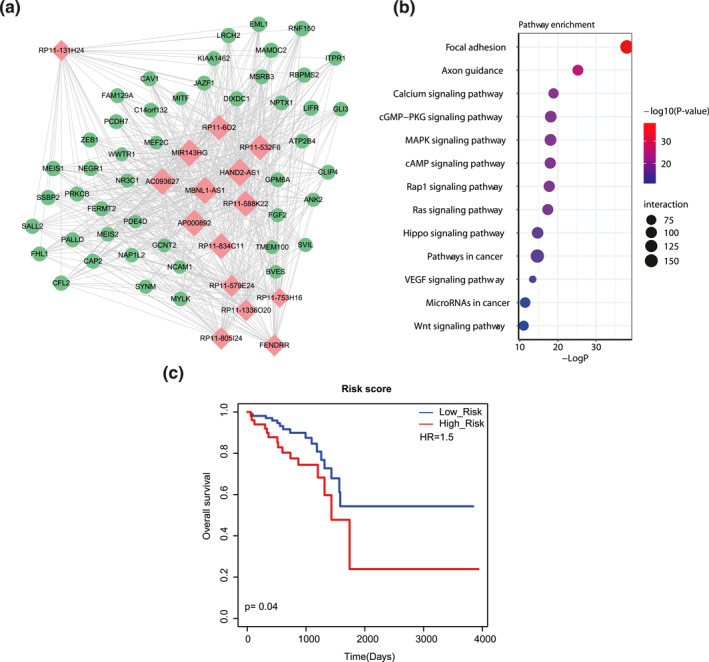
Extraction and analysis of a hub subnetwork. (a) Visualization of the hub subnetwork. Red nodes represent lncRNAs and green nodes represent mRNAs. (b) Pathway enrichment of the mRNAs of the hub subnetwork. Node size represents the number of overlapped genes between the mRNAs in subnetwork and pathways. Node colour represents statistical significance of pathway enrichment. (c) Kaplan–Meier survival curves of the hub subnetwork

According to the above results, we have known that the nodes with larger degrees usually represented greater importance in the scale‐free network. So, it was necessary to look for nodes with largest degrees (namely hub nodes) and conduct in‐depth analysis. In this study, top 10% lncRNAs and top 5% mRNAs with the largest degrees were chosen together as hub nodes. Then, all these hub lncRNAs, hub mRNAs and the interactions between them were extracted from the rectal cancer‐related lncRNA‐mRNA network for constructing a hub subnetwork (Figure 4a). Excitingly, we could see that some genes of the subnetwork were reported to be associated with rectal cancer. For example, *PRKCB* was found to be involved in a biomarker model, which could predict rectal cancer response to preoperative chemoradiotherapy [24]. The cell surface protein *NCAM1*, also known as *CD56*, was one of the most specific proteomic markers for natural killer cells. Tackling a natural killer‐like response after therapy may improve outcomes of rectal cancer patients [25]. By improving epithelial‐mesenchymal transition in a *ZEB1*‐dependent manner, *OCT4* could enhance radio‐resistance development in rectal cancer cells [26]. *PCDH7* was known to be involved in the regulation of cell adhesion, which could regulate programed cell death, cell proliferation and cell‐cell communication [27]. Caveolin‐1 (*Cav1*) was implicated in tumour cell migration and metastasis, which was validated as an independent predictor of decreased survival in rectal cancer [28]. Significant down‐regulation of *FGF2* was observed in the post ganetespib treatment of rectal cancer patients [29]. More importantly, we found some novel lncRNAs at the core status of the hub subnetwork, such as *RP11‐1336O20* and *HAND2‐AS1* (Figure 4a). These two lncRNAs were also demonstrated as DE nodes that had significant prognosis capability in the above analysis. Thus, we thought these lncRNAs might be novel biomarkers in the future study of rectal cancer. Further pathway enrichment results showed that these genes were significantly enriched in the well‐known cancer‐related pathways, such as Focal adhesion, Pathways in cancer and Wnt signalling pathway (Figure 4b). In addition, we calculated the risk score using linear combination of expression values of lncRNAs of the subnetwork weighted by the regression coefficient of univariate Cox regression and divided the rectal cancer patients into high‐risk group and low‐risk group. By performing Kaplan–Meier survival analysis, the results showed that high‐risk group and low‐risk group of rectal cancer patients were significantly distinguished by different clinical outcomes (Figure 4c, *p* = 0.04). All the above results showed the efficiency of information transport through the rectal cancer‐related hub subnetwork.

### 3.4 Identification of rectal cancer‐related functional modules

**FIGURE 5:**
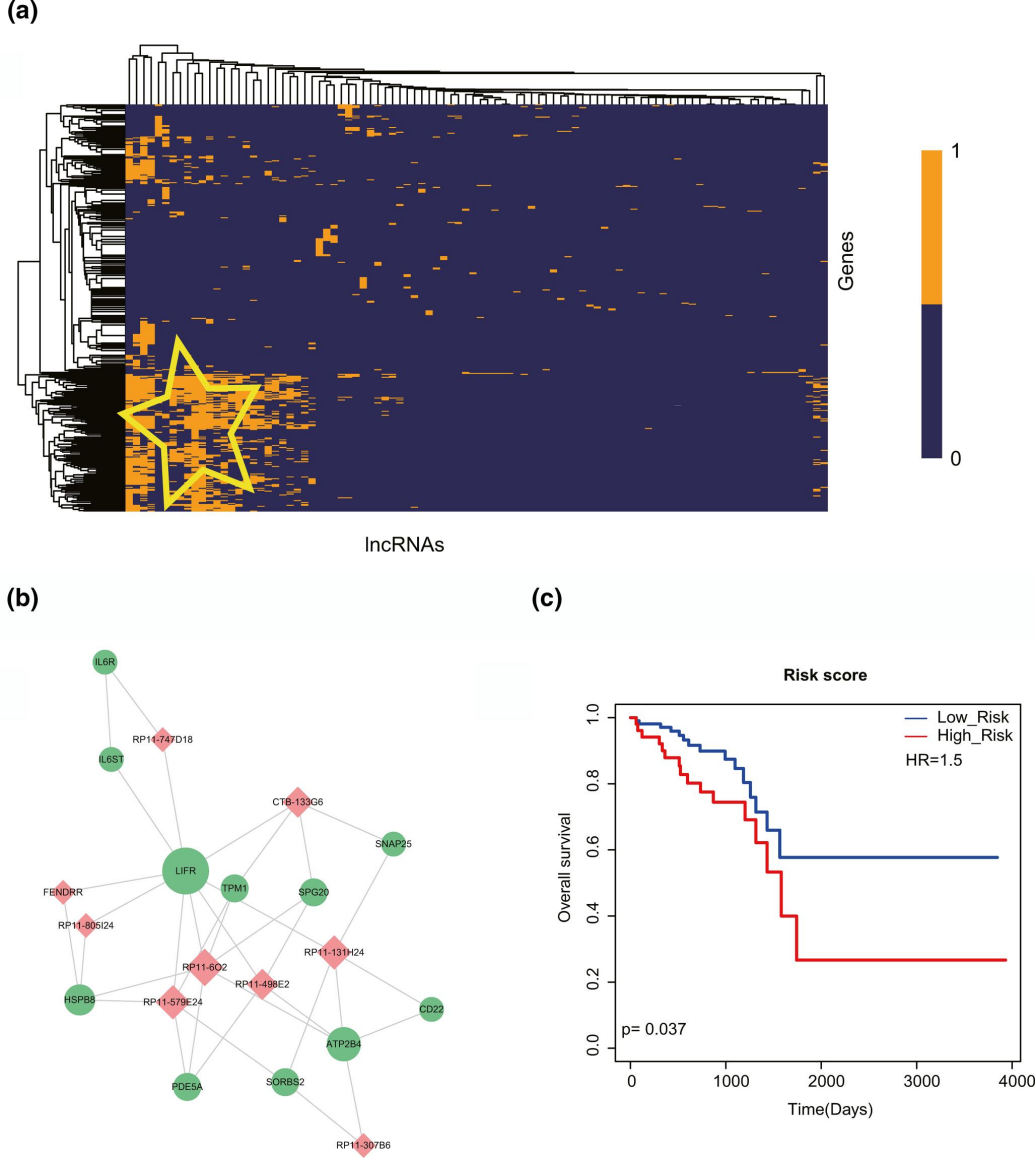
Identification and analysis of the functional module. (a) The heat map of hierarchical clustering of all the lncRNAs and mRNAs of the rectal cancer‐related lncRNA‐mRNA network. Yellow region represents the lncRNAs and mRNAs that have a distinct tendency of co‐expression. (b) Visualization of the functional module. Red nodes represent lncRNAs and green nodes represent mRNAs. (c) Kaplan–Meier survival curves of the functional module

Our rectal cancer‐related lncRNA‐mRNA network has been validated with larger average cluster coefficient and shorter average path length. This phenomenon suggested the importance of identifying local modules. We first performed hierarchical clustering to all the lncRNAs and mRNAs of the rectal cancer‐related lncRNA‐mRNA network. Interestingly, some lncRNAs and mRNAs were found to have a distinct tendency of co‐expression (Figure 5a). Finding biologically significant modules was the major goal of co‐expression analysis. Therefore, MCODE was next used to screen a rectal cancer‐related functional module from the lncRNA‐mRNA network. As a result, the module was a cluster of highly correlated nodes, containing nine lncRNAs and 11 mRNAs (Figure 5b). Actually, given a network of interacting genes, modules were typically identified as ‘hot spots’, which were often associated with a disease outcome [30]. To test whether the identified module was biologically meaningful, we calculated the risk score for each rectal cancer patient by linear combination of the expressional value of each node weighted by the regression coefficient derived from the univariate Cox model; in this way, patients could be divided into high‐risk group and low‐risk group with the median risk score as threshold. By using Kaplan–Meier estimation and log‐rank test, it was indicated that a significant difference of survival outcomes was observed between the high‐risk group and low‐risk group (Figure 5c, *p* = 0.037). From the results mentioned above, we concluded that a series of RNAs contained in the functional module identified from the whole network could be used as novel biomarkers to predict the prognosis of the rectal cancer patients, which showed a potential possibility in clinical applications.

### 3.5 Identification of core TF‐lncRNA crosstalk

**FIGURE 6:**
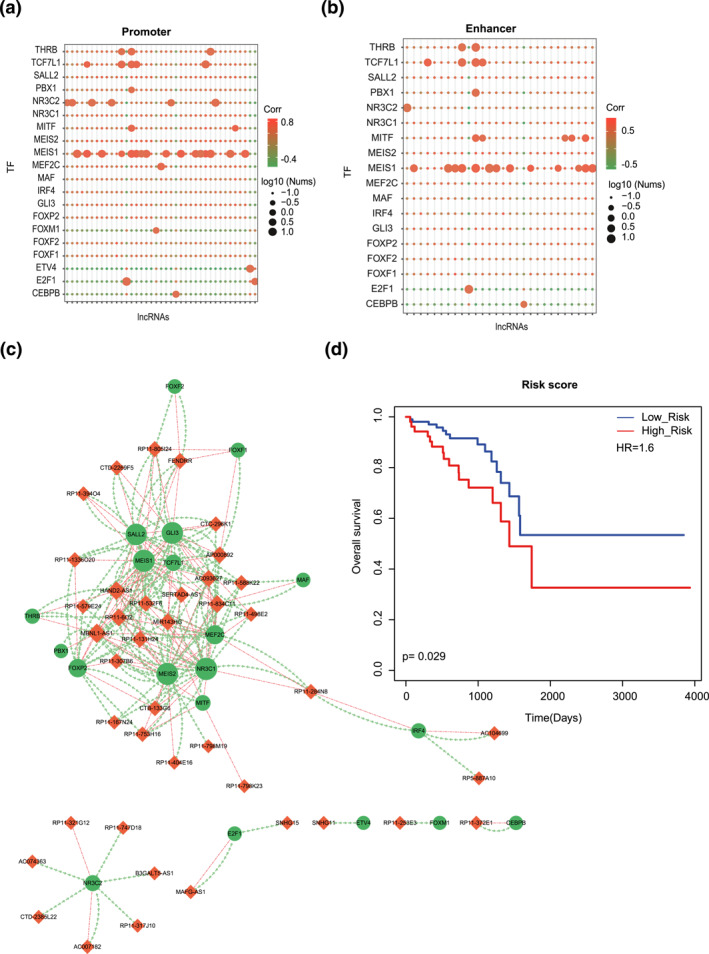
Identification of core TF‐lncRNA crosstalk based on motif analysis. (a) TF motif searching of promoter regions of lncRNAs. Node colour represents the correlation score of PCC. Node size represents the number of TFs that bind to the promoter regions of lncRNAs. (b) TF motif searching of enhancer regions of lncRNAs. Node colour represents the correlation score of PCC. Node size represents the number of TFs that bind to the enhancer regions of lncRNAs. (c) Visualization of the core TF‐lncRNA crosstalk network. Orange nodes represent lncRNAs and green nodes represent TFs. Orange lines represent TFs binding to the promoter/enhancer regions of lncRNAs. Green lines represent ceRNA relationships between TFs and lncRNAs. (d) Kaplan–Meier survival curves of the core TF‐lncRNA crosstalk network

In our study, we mainly constructed the rectal cancer‐related lncRNA‐mRNA network based on the ceRNA theory. That is, lncRNAs could regulate mRNAs from the perspective of ceRNA network. In addition, the previous studies have validated that TFs could regulate the expression of lncRNAs by binding to their DNA regulatory elements [31]. Thus, at the end of the study, we explored the crosstalk between TFs and lncRNAs. To do this, we first downloaded TFs from AnimalTFDB (http://bioinfo.life.hust.edu.cn/AnimalTFDB/#!/) and mapped them into the lncRNA‐mRNA network for screening lncRNA‐TF interactions. Then, we defined the promoter region of an lncRNA as a basal domain of −2 kb to +2 kb around the transcriptional start site (TSS). We also downloaded enhancer regions from FANTOM5 project [32]. The enhancer region of lncRNA was defined if it was located in the domain with more than ±2 kb from the TSS. Motif, summarizing the collection preferentially bound of certain TF [33], was obtained by using Find Individual Motif Occurrences (FIMO) [34]. As a result, the potential correlations based on the biological regulation of lncRNAs transcription exerted by TF through its binding to the specific motif of promoter and enhancer with a threshold of FIMO *p*‐value <1e‐4 were displayed, respectively (Figure 6a and b). These results confirmed that these lncRNAs and TFs had both ceRNA and motif binding relationships, which explained the crosstalk between TFs and lncRNAs. Then, we integrated all these significant lncRNA‐TF ceRNA pairs through motif enrichment to construct a core lncRNA‐TF crosstalk network (Figure 6c). Considering these nodes as a gene set, we performed survival analysis by univariate Cox regression. The results showed that patients in high‐risk group and low‐risk group of rectal cancer patients could be significantly distinguished with different clinical outcomes (Figure 6d, *p* = 0.029). These results may uncover potential regulatory mechanisms between ceRNA pairs in our network and indicate its potential predictive capability for prognostic outcomes.

## 4 DISCUSSION

Rectal cancer is a malignant tumour and has poor prognosis. It is urgent to identify new therapeutic regulators to elucidate the molecular mechanisms and biological functions with the purpose of improving the survival outcomes of rectal cancer patients. Cancer is considered as a system of multi‐gene expression patterns. Recently, the increasing studies have paid attention to the application of lncRNAs in cancer research because lncRNAs could play a wide variety of roles in the process of carcinogenesis and cancer metastasis [35]. As an important aspect of lncRNA research, lncRNA‐mediated ceRNA regulatory network has been proposed to shed light on the mechanism of tumourigenesis and disease progression of rectal cancer. For example, PVT1, which is highly up‐regulated in rectal cancer cells and tissues, functions as a ceRNA in rectal cancer via the PVT1/miR‐30d‐5p/RUNX2 axis to promote cell proliferation and invasion [36]. Similarly, Cui et al. found that UCA1 functions as the ceRNA of tumour suppressor miR‐28‐5p and thereby hinders the expression of HOXB3, which promoted cancer cell proliferation and invasion [37]. Hao et al. indicated that lncRNA SNHG15 binds to a fast‐turnover transcription factor Slug to rectal cancer cell invasion and metastasis [38]. Thus, we aimed to explore the biologically regulatory mechanism under the lncRNA‐mediated ceRNA framework to suggest valuable research directions for the diagnosis and treatment of rectal cancer.

In this study, with comprehensive analysis of the lncRNA/mRNA expression datasets, obvious correlations of expressions were discovered between the DE lncRNAs and DE mRNAs. Especially, some lncRNAs were highly correlated with multiple DE mRNAs, such as *HAND2‐AS1*, *RP11‐167N24* and *RP11‐1336O20*, which might demonstrate a further research significance. Owing to the great importance of lncRNAs in the study of rectal cancer, we focussed on further computational analysis of lncRNAs under the background of ceRNA network.

A rectal cancer‐related DE lncRNA‐DE mRNA network was constructed based on ceRNA hypothesis. Topology features including larger average cluster coefficient, shorter average path length and power law distribution indicated the network meet the characteristics of a small world. However, biological network was often too large to display local structure for detailed information. Most genes in a network were not oncogenes but noise signals [39]. Therefore, hub lncRNAs/mRNAs with larger degrees in the rectal cancer‐related lncRNA‐mRNA network were further chosen to construct a hub subnetwork. According to literature validation, we detected some rectal cancer‐related mRNAs including *PRKCB*, *NCAM1*, *ZEB1*, *PCDH7*, *Cav1* and *FGF2* in the subnetwork. For instance, Cho et al. built a multi‐gene mRNA model using PRKCB and other related mRNAs, which showed a good ability (AUC:0.846) to predict the response of preoperative chemoradiotherapy for rectal cancer [24]. *NCAM1*, also termed CD56(+), was found to be increased in colorectal cancer patients after preoperative nutritional support, which might indicate a positive effect on the prognosis [40]. Yingmin et al. discovered that lncRNA *RP11‐138 J23*.*1* could prevent the proteasomal degradation of *ZEB1*, which promoted the development of rectal cancer both in vitro and in vivo [41]. Mateusz et al. detected the decreasing expression of *PCDH7*, known for its involvement in the intercellular connections, in the colorectal tumour tissues. High expression level of *CAV1* was usually correlated with tumour progression [42], while Juanli et al. found that the overexpression of *Cav1* could weaken the proliferation and invasion abilities of rectal cancer cells through inhibiting activation of *EGFR* [43]. Furthermore, by building 2D and 3D co‐culture models, Sarah et al. indicated *FGF2* was correlated with the rectal cancer cell migration and invasion, and blocking the signalling of *FGF2* would suppress these abilities [44]. More interestingly, some novel lncRNAs such as *MBNL1‐AS1* and *HAND2‐AS1* were found at the core region of the hub subnetwork. Kongxi et al. found that LncRNA *MBNL1‐AS1* could competitively bind to the *microRNA‐412‐3p* to suppress the rectal cancer cell metastasis and invasion via regulating *MYL9* [45]. For example, Jianwei et al. found that the expression of lncRNA *HAND2‐AS1* was significantly low in the rectal cancer tissues, and *HAND2‐AS1* could sponge *miR‐1275* by targeting *KLF14* to inhibit tumour propagation in vivo [46]. Zhipeng et al. indicated that *HAND2‐AS1* could regulate the expression of *PDCD4* by sponging *miR‐20a* to reverse 5‐fluorouracil resistance in colorectal cancer [47]. By the articles mentioned above, we thought they could be potential biomarkers for tumourigenesis and progression of rectal cancer. Besides, a functional module was identified from the large lncRNA‐mRNA network by MCODE, which was validated as an important approach to analyse high‐dimensional biological datasets [48]. It seems that the modules outperform gene signatures in predicting disease prognosis and indicating cellular function. In addition to the ceRNA theory, it is worth noting that TFs could regulate the expression of lncRNAs by binding to their DNA regulatory elements. Thus, a core lncRNA‐TF crosstalk network was finally identified to indicate how the TFs regulated the expressions of the lncRNAs, which simultaneously formed a ‘feedback loop’ to uncover the inner crosstalk mechanisms between the lncRNAs and TFs.

Based on all the above analyses, we identified core lncRNAs and lncRNA‐mRNA crosstalks in the network, hub subnetwork, functional module and lncRNA‐TF crosstalk network, which function in the rectal cancer‐related biological process and molecular mechanism. To further explain the specific prognostic biomarkers for long‐term survival of rectal cancer patients, all these elements were used for survival analysis. The results showed that all the core lncRNAs, hub subnetwork, functional module and crosstalk network could significantly distinguish the high‐risk group and low‐risk group of rectal cancer patients. Generally, in this study, we constructed the lncRNA‐mRNA networks and performed topological analyses. First, we extracted the hub gene‐associated subnetwork, which was considered as the core of the network. We used the risk score model to test the prognostic effects of the subnetwork (Figure 4c). Second, we identified the network modules based on network topology and tested the prognostic effects of the module (Figure 5c). Third, we integrated lncRNA‐TF ceRNA pairs and TF binding affinities to identify lncRNA‐TF positive feedback loops and tested the prognostic effects of the feedback loops (Figure 6d). Although the results showed that the prognostic effect of feedback loops was better than that of the other two models, we considered that all the three models showed good prognosis performances and were equally important.

There are also some limitations in our analysis. First, a series of biological analysis was conducted to identify some important factors during the regulatory process of the transcription, but it is better to validate certain functions and mechanisms of some hub genes through experiments. Second, if we measured the hub genes via a stricter screening cut‐off, more biologically significant loci might be found to point the way for further research. Third, we download the sequences of TFs from the FANTOM5 database and mapped them to the lncRNAs‐mRNAs ceRNA network established on the basis of TCGA, which might reduce the accuracy of the predictive crosstalk between TFs and lncRNAs. Fourth, in this study, we also downloaded the miRNA expression from TCGA portal and aimed to identify mRNA‐miRNA‐lncRNA triple ceRNA network. But the sample numbers of miRNA profile was not matched to the mRNA or lncRNA profiles (Sample size: 92 vs. 177). We thought that the unmatched sample numbers could lead to a bias result. Furthermore, we also validated the clinical values in other datasets, the results showed that a multiple gene model (Figure 2) was also significant in GSE 133057 (Supplementary Figure S2). However, the rectal cancer dataset was not sufficient and the sample scale was small. This was also a limitation in our study. In addition, healthy control samples are limited in the TCGA dataset. The unbalanced sample size might reduce the robustness and the reliability of the model. In the future, we will perform our model in the newly released data of READ or perform our model in the GTEx‐TCGA integrated dataset. A cancer sample‐specific lncRNA‐mRNA network will also be constructed to investigate the regulatory mechanisms in cancers.

In a word, our study provided potential prognostic biomarkers and valuable thoughts for further rectal cancer studies, which was worthy of experimental validations.

## CONFLICT OF INTEREST

The authors made no disclosures.

## Supporting information

Supplementary Material

Supplementary Material

Supplementary Material

## Data Availability

The data that support the findings of this study are available from the corresponding author upon reasonable request.
