# Supplementary figures and images for: Construction and characterization of rectal cancer‐related lncRNA‐mRNA ceRNA network reveals prognostic biomarkers in rectal cancer
Source: IET Syst Biol. 2021 Oct 6;15(6):192–204. doi: 10.1049/syb2.12035 (PMC8675822; doi:10.1049/syb2.12035)

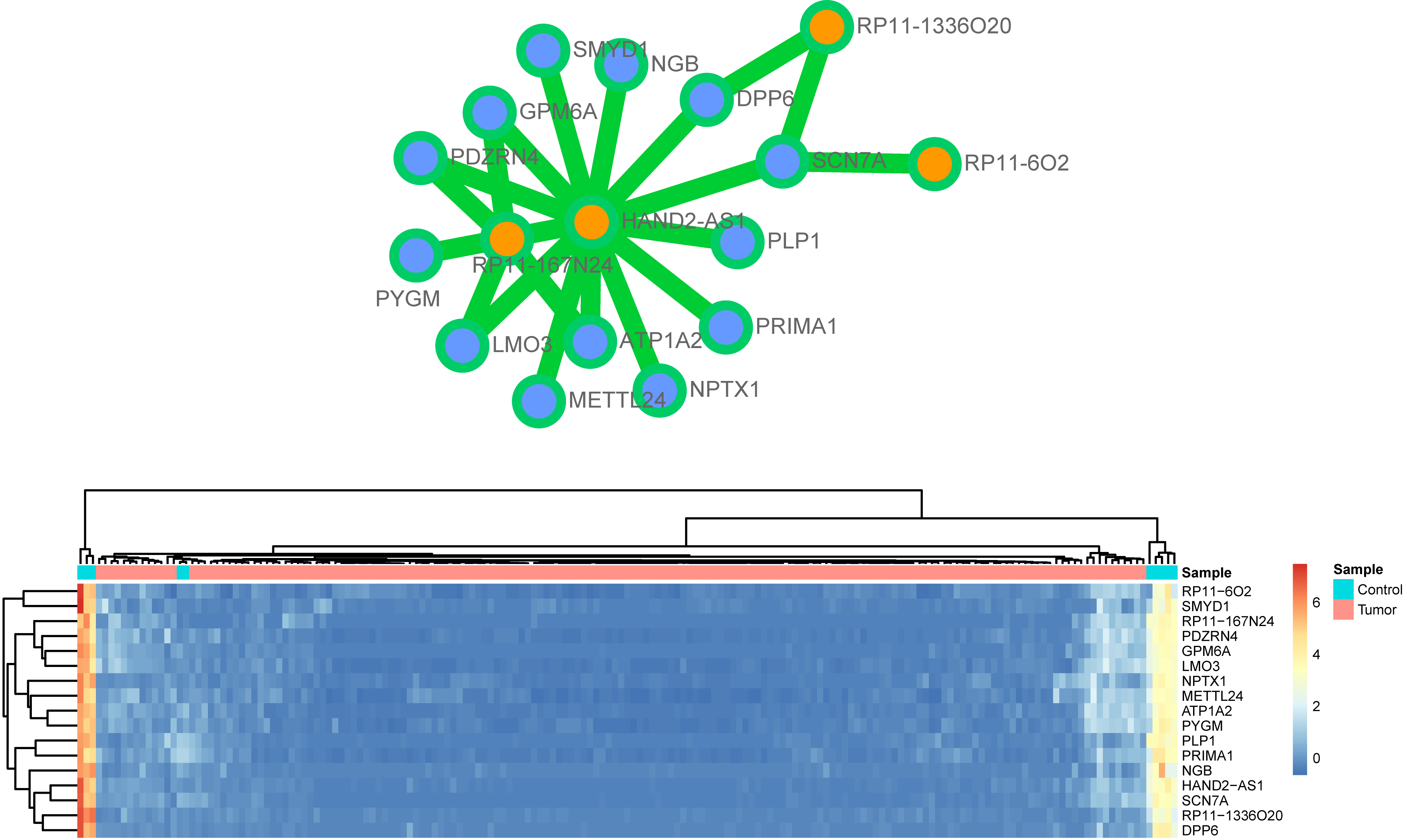

Supplement: Supplementary file 2 — Supplementary Material [file SYB2-15-192-s001.tif]

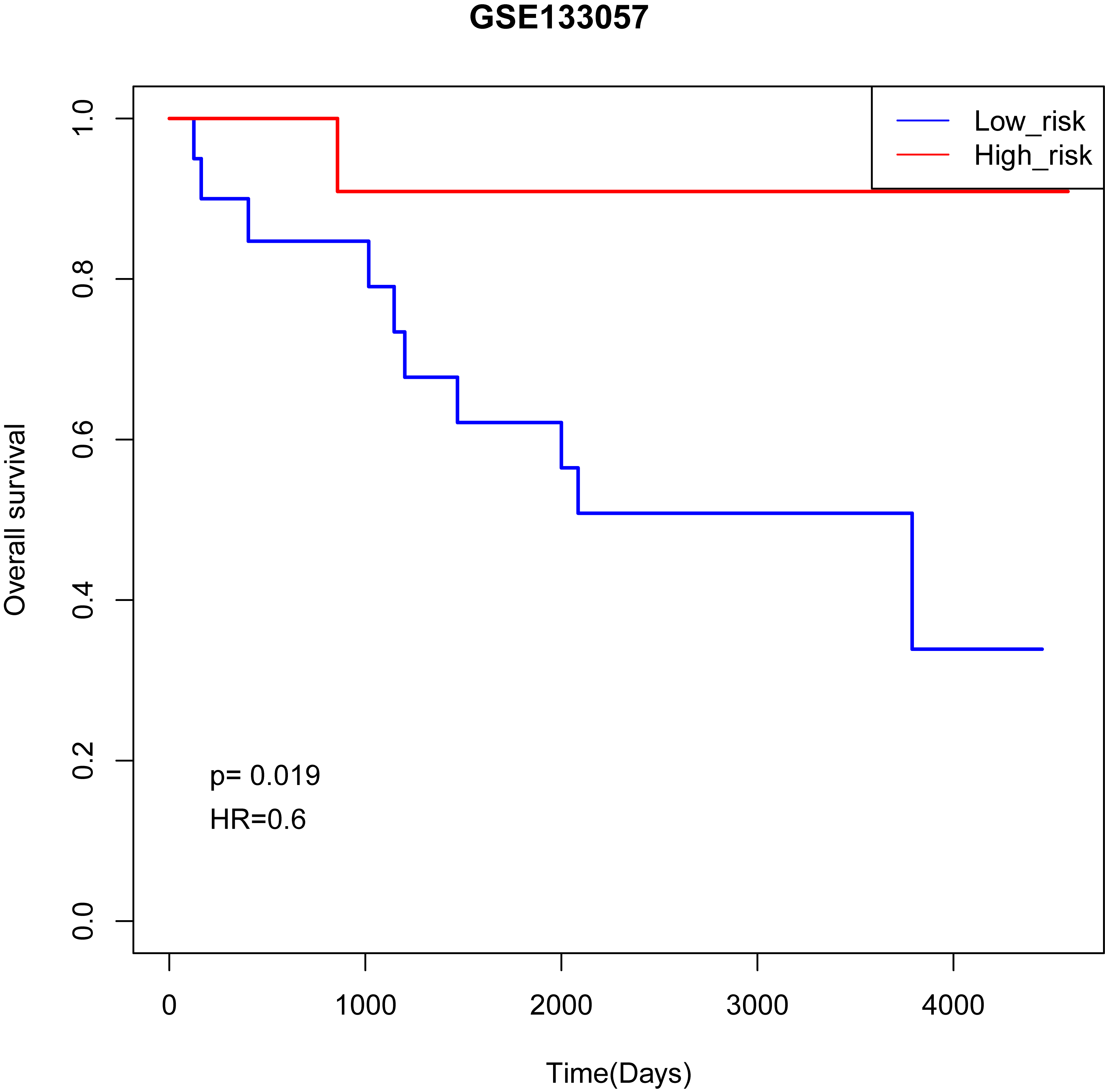

Supplement: Supplementary file 3 — Supplementary Material [file SYB2-15-192-s003.tif]
